# Ultraviolet Irradiation Induces the Accumulation of Chondroitin Sulfate, but Not Other Glycosaminoglycans, in Human Skin

**DOI:** 10.1371/journal.pone.0014830

**Published:** 2011-08-04

**Authors:** Benjamin Boegel Werth, Muhammad Bashir, Laura Chang, Victoria P. Werth

**Affiliations:** 1 Medical Research, Philadelphia Veterans Administration Medical Center, Philadelphia, Pennsylvania, United States of America; 2 Department of Dermatology, University of Pennsylvania, Philadelphia, Pennsylvania, United States of America; Tufts University, United States of America

## Abstract

Ultraviolet (UV) light alters cutaneous structure and function. Prior work has shown loss of dermal hyaluronan after UV-irradiation of human skin, yet UV exposure increases total glycosaminoglycan (GAG) content in mouse models. To more fully describe UV-induced alterations to cutaneous GAG content, we subjected human volunteers to intermediate-term (5 doses/week for 4 weeks) or single-dose UV exposure. Total dermal uronyl-containing GAGs increased substantially with each of these regimens. We found that UV exposure substantially increased dermal content of chondroitin sulfate (CS), but not hyaluronan, heparan sulfate, or dermatan sulfate. UV induced the accumulation of both the 4-sulfated (C4S) and 6-sulfated (C6S) isoforms of CS, but in distinct distributions. Next, we examined several CS proteoglycan core proteins and found a significant accumulation of dermal and endothelial serglycin, but not of decorin or versican, after UV exposure. To examine regulation in vitro, we found that UVB in combination with IL-1α, a cytokine upregulated by UV radiation, induced serglycin mRNA in cultured dermal fibroblasts, but did not induce the chondroitin sulfate synthases. Overall, our data indicate that intermediate-term and single-dose UVB exposure induces specific GAGs and proteoglycan core proteins in human skin in vivo. These molecules have important biologic functions and contribute to the cutaneous response to UV.

## Introduction

Ultraviolet light is a major environmental source of damage to skin. A hallmark of chronically photodamaged skin is the induction of glycosaminoglycans (GAGs) in the dermis [1]. GAGs are unbranched, polyanionic polysaccharide chains made up of repeating disaccharide units. The GAGs of normal skin include chondroitin sulfate (CS), hyaluronic acid (HA), dermatan sulfate (DS), heparan sulfate (HS), and keratan sulfate [2], each of which exerts distinct biologic actions. All GAGs, except HA, are covalently attached to a polypeptide backbone called a core protein, and the resulting protein-carbohydrate complex is called a proteoglycan (PG). Much of the GAG content within chronic solar elastoic lesions consists of CS linked to the versican core protein [3], [4]. However, there has been no systematic study of the alterations to GAGs or PGs during the early stages of photodamage in human skin. The SKH-1 hairless mouse has been used to model the increase in cutaneous GAGs in response to UV exposure [5], [6], but different published reports describe increases in CS [7], DS [8], HA [8], or HS without an increase in DS [9]. Moreover, two recent studies indicate that HA actually decreases in response to acute [10] and chronic [11] UV irradiation.

In the past, the function of GAGs and proteoglycans was assumed to be primarily structural. Indeed, GAGs are major structural components of the skin's extracellular matrix and important in storage of water and electrolytes [12], [13]. Recent literature suggests that specific GAGs have roles in inflammatory processes [14], [15].

In the current study, we re-examined the cutaneous response to UV by determining if short- and intermediate-term UV exposure of human skin induces accumulation of GAGs, as assessed by Hale stain, and then used specific antibodies and mRNA probes to characterize specific molecular species of GAGs and PGs. Importantly, the responses of human skin during these early periods have not been previously characterized, nor have the effects of specific wavelengths of light. Knowledge of the responses of specific GAGs and PGs to UV could further our understanding of the early events that lead to photodamage. Therefore, in the current study, we determined if short- and intermediate-term UV exposure induced accumulation of GAGs, as assessed by Hale stain, and then used specific antibodies and mRNA probes to characterize specific molecular species involved in the response to UV.

## Results

### Intermediate-term UVB irradiation increases total dermal GAG content of human skin in vivo

**Figure 1:**
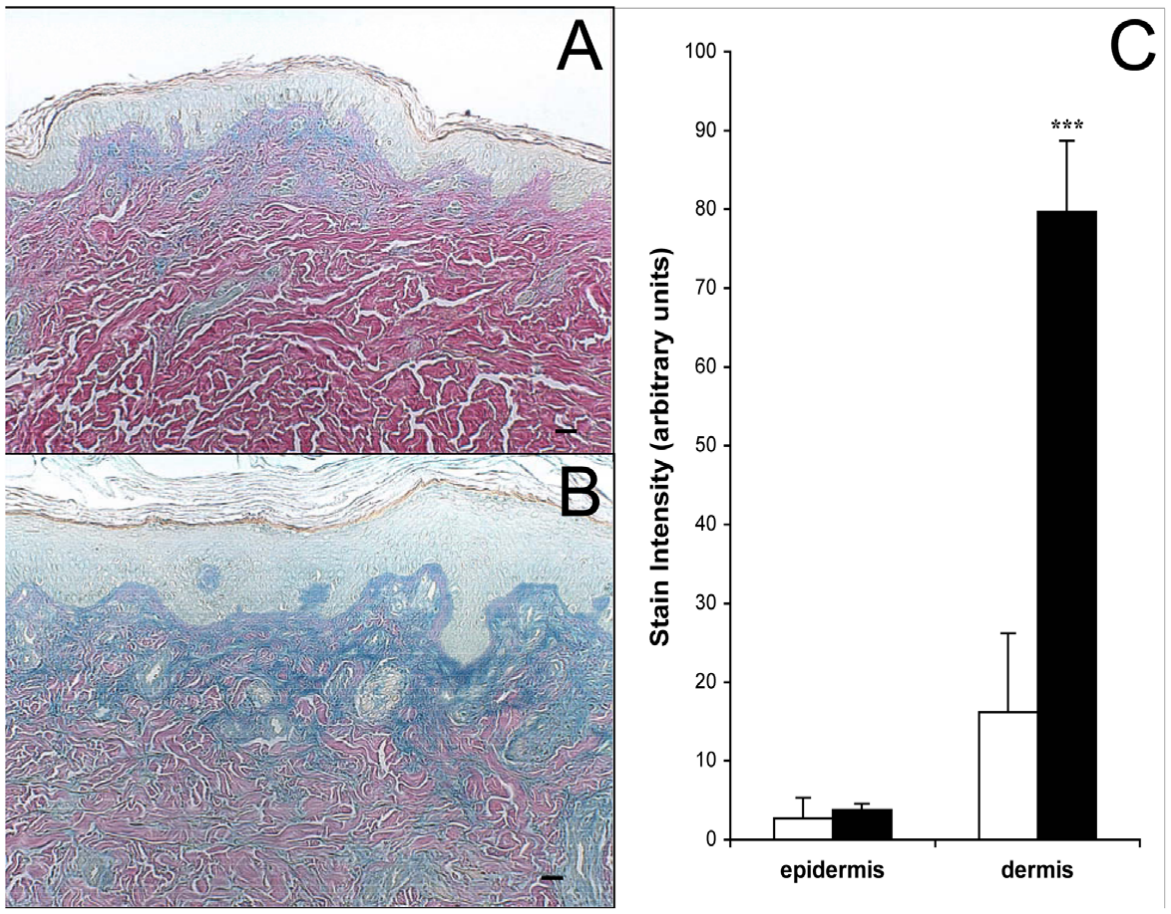
Effect of intermediate-term UVB irradiation on total dermal GAG content of human skin in vivo. Human volunteers were sham-irradiated or exposed to UVB 5 times weekly for 4 weeks. Biopsies were taken and GAGs were visualized using Hale's stain. Collagen stains red, cytoplasm stains yellow, and GAGs stain blue in (a) control skin and (b) UVB-irradiated skin. The images in this figure are of tissue from the same patient. The GAG stain intensity was quantified using ImagePro version 3 and graphed (c). Sham skin is represented by open bars and UVB-irradiated skin by solid bars. (size bars = 62.5 µm, ***p<.001 versus sham, n = 5).

Four weeks of UVB exposure, delivered in five doses per week to healthy human volunteers, resulted in a striking increase in GAG content, indicated by blue staining by Hale, in irradiated relative to non-irradiated skin. Control skin shows localization of GAGs below the dermal-epidermal junction (Figure 1a). Epidermal GAG staining with Hale was substantially lighter than dermal staining in both control and UVB-exposed skin, and irradiation did not significantly change it. Quantitative image analyses showed that the dermis of UVB-irradiated skin stained 4.9 times more intensely for GAGs than the dermis of non-irradiated skin (Figure 1b,c). Visual inspection indicated that the distribution of dermal GAGs was altered by UVB exposure. In normal tissue, GAGs are concentrated in the upper dermis close to the dermal-epidermal junction (Figure 1a). UVB-irradiated skin exhibits GAGs deeper in the dermis, although they are still most densely localized directly below the epidermis (Figure 1b). There was no accumulation of elastosis seen in these intermediate-term irradiation experiments done on non sun-exposed skin (data not shown).

### Intermediate-term UVB irradiation of human skin in vivo does not significantly alter dermal content of HA, DS, or HS

**Figure 2:**
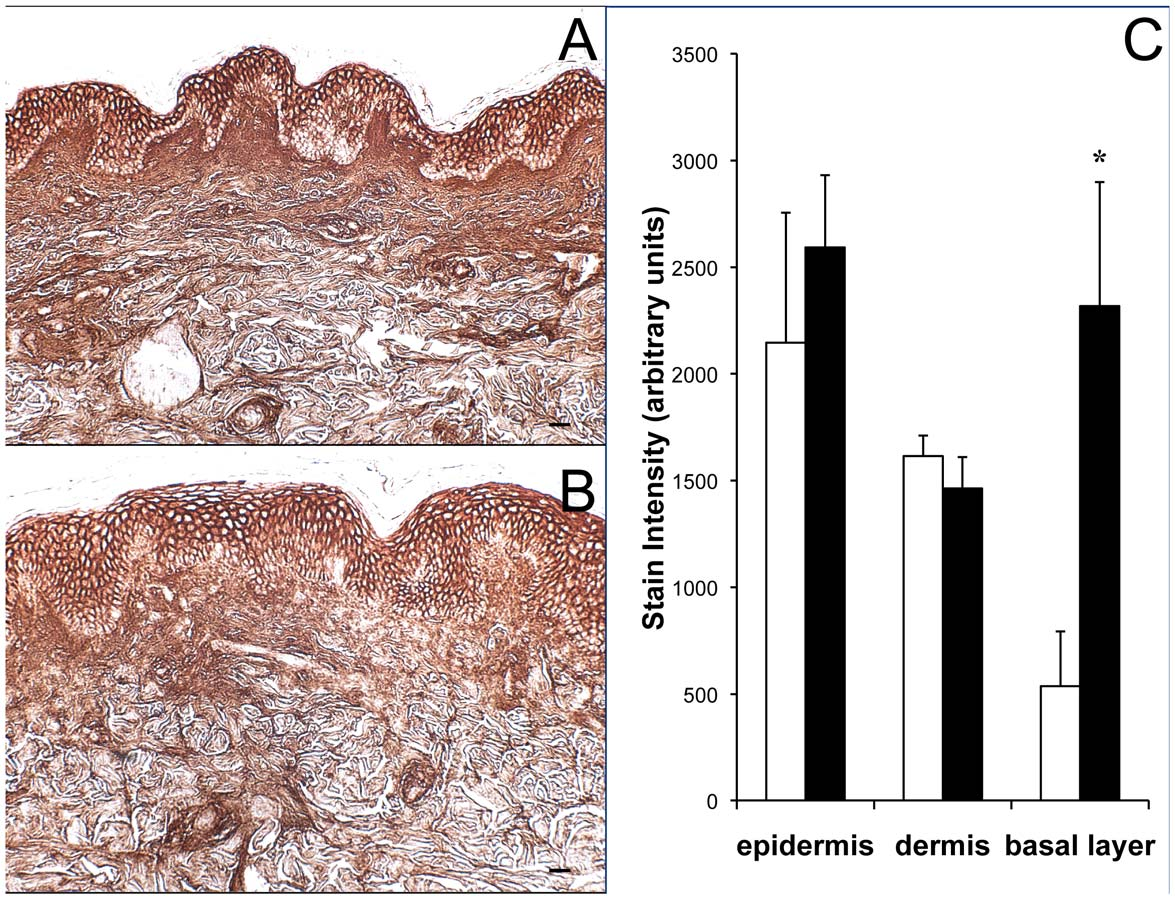
Effect of intermediate-term UVB exposure on dermal HA in vivo. Skin was (a) sham-irradiated or (b) UVB-irradiated 5 times weekly for 4 weeks. HA was labeled with biotinylated HABP and visualized using a peroxidase system. The images in this figure are of tissue from the same patient. Stain intensity in dermis, epidermis, and the basal layer of the epidermis was quantified using ImagePro version 3 and graphed (c). Sham skin is represented by open bars and UVB-irradiated skin by solid bars. (size bars = 62.5 µm, *p<.05).

HA, a uronic acid-containing GAG, was visualized using biotinylated HA binding protein (HABP), visualized with a streptavidin horseradish peroxidase system. Normal human skin displayed HA throughout the dermis (Figure 2a). Dark staining was evident in the upper epidermis of non-exposed skin, but the basal layer did not contain much HA. UVB exposure resulted in HA occupying the basal layer as well as the rest of the epidermis, with no significant change in the total HA content of the epidermis (Figure 2b,c). HABP staining in the upper dermis of UVB-exposed skin was more diffuse than that in the upper dermis of normal skin, but UVB did not cause significant reduction in the overall dermal HA content (Figure 2b,c). IHC using a monoclonal anti-HS and anti-DS antibody showed no UVB induction of HS or DS (data not shown).

### Intermediate-term UVB irradiation increases dermal CS content of human skin in vivo

**Figure 3:**
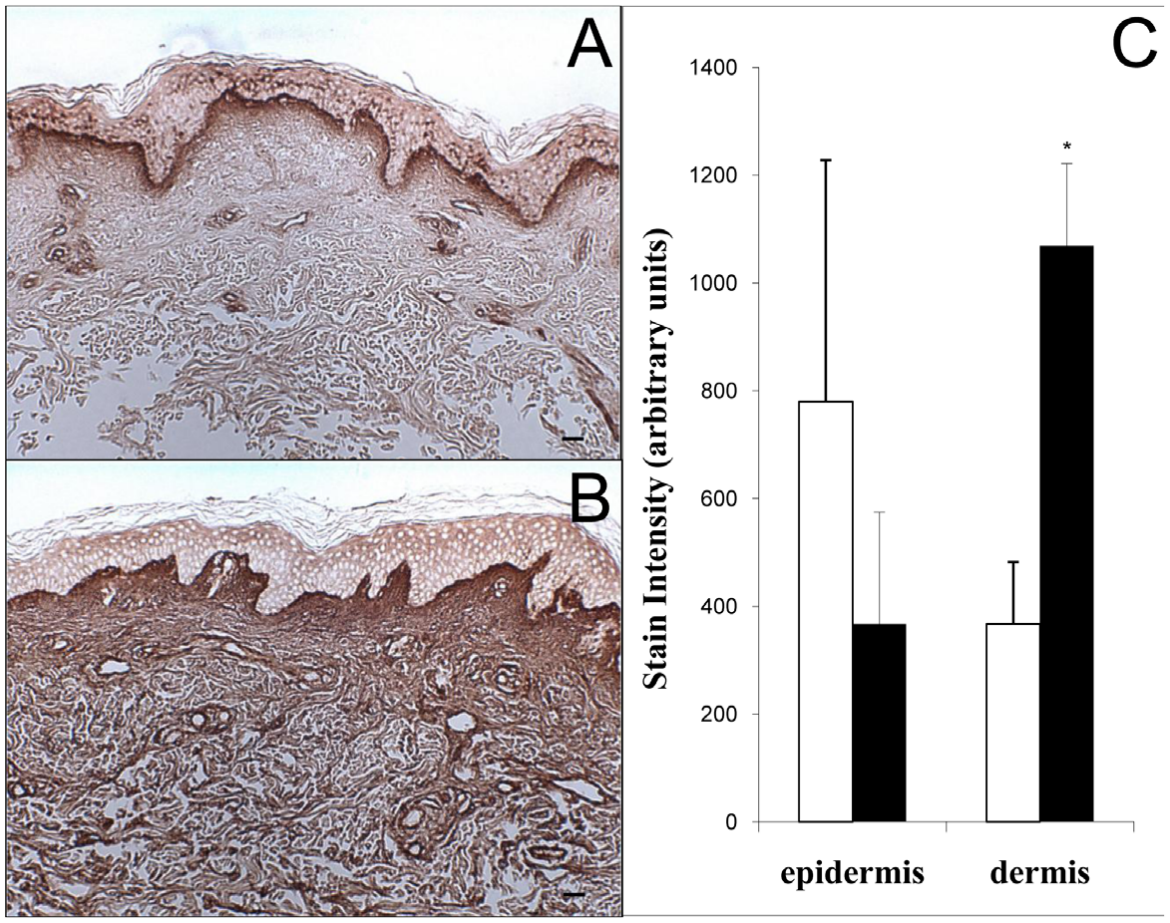
Localization and quantification of CS in human skin following intermediate-term UVB irradiation. Skin was (a) sham-irradiated or (b) UVB-irradiated 5 times weekly for 4 weeks. CS was labeled with the CS-56 anti-CS antibody and visualized using a biotin-peroxidase system. The images in this figure are of tissue from the same patient. Stain intensity in the dermis was quantified using ImagePro version 3 and graphed (c). Sham skin is represented by open bars and UVB-irradiated skin by solid bars. (size bars = 62.5 µm, *p<.05).

Chondroitin sulfate was detected using monoclonal antibody CS-56, which recognizes C4S and C6S, but not dermatan sulfate. In normal skin, epidermal staining was variable but typically present (Figure 3a). The great majority of CS is localized in the dermis, in a dense band directly below the dermal-epidermal junction. With chronic UVB exposure, epidermal CS content is variable and not induced (Figure 3b). UVB irradiation results in CS content deeper in the dermis, and quantification of dermal stain intensity shows an increase to nearly three times the unirradiated controls (Figure 3c). CS staining intensity data for the epidermis was not included because epidermal staining was faint and variable, and did not change with UVB. Because CS accumulates in the same locations where blue Hale staining increases, but HA, HS, and DS do not, CS appears to be largely responsible for the UVB-induced increase in GAG content of human skin.

### Intermediate-term irradiation of human skin in vivo with different UV wavelengths increases dermal content of total GAGs and CS

**Figure 4:**
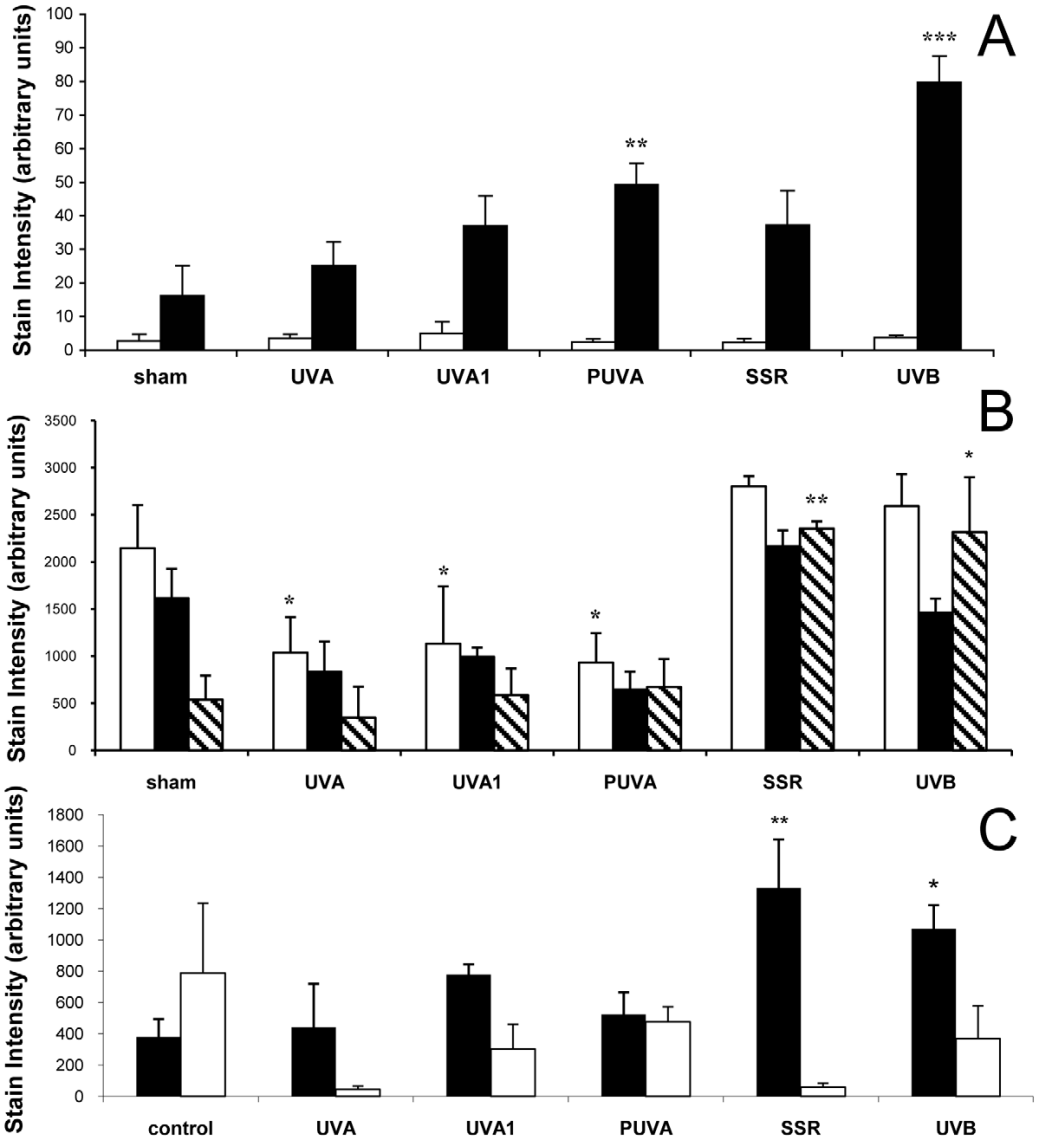
Wavelength-specific effects of intermediate-term irradiation on GAGs, HA, and CS. Normal human volunteers were treated with sham, UVA, UVA1, PUVA, SSR, and UVB on different sections of their backs 5 times weekly for 4 weeks. ImagePro 3 analysis of treatment-specific effects were evaluated for (a) total GAG content with Hale stain, (b) HA, and (c) CS. Open bars represent epidermal stain intensity, solid bars represent dermal stain intensity, and hatched bars represent stain intensity in the basal layer of the epidermis. (***p<.001, **p<.01, *p<.05, n = 5 for Figure 4a, n = 4 for Figures 4b-c).

To determine wavelength specificity, we repetitively exposed subjects to UVA, UVA1, PUVA, and SSR as well as UVB. UVB caused the greatest increase over control in total dermal GAG content as detected by Hale stain, and PUVA was the only other irradiation method that also produced a significant increase (Figure 4a). UVB was previously shown to be more effective than UVA at inducing GAG accumulation in SKH1 hairless mouse skin [16]. To examine specific GAG species, we found that the HA content of human dermis was not significantly altered by any treatment (Figure 4b). UVA, UVA1, and PUVA significantly reduced epidermal HA (Figure 4b). UVB and SSR caused significant increases of HA in the basal layer of the epidermis. The amount of dermal CS was significantly increased over non-irradiated skin by UVB and by SSR (Figure 4c). CS staining in the epidermis was generally faint and variable, and did not change significantly with any UV treatment (Figure 4c).

### Single-dose UVB irradiation in vivo alters the HA content of human skin

**Figure 5:**
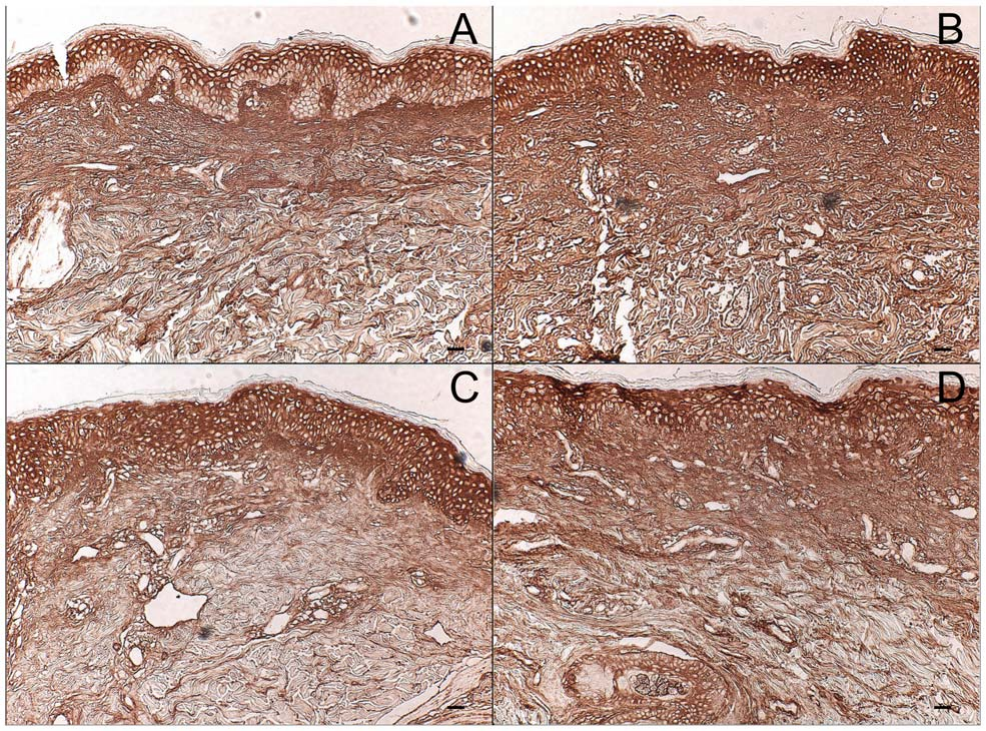
Time course for changes in HA content of UVB-irradiated skin. Normal human volunteers were exposed to 2X MED of UVB. Biopsies were taken 1, 2, and 3 days after irradiation. HA was labeled with biotinylated HABP and visualized using a peroxidase system. Shown are stains of (a) control tissue and tissue taken (b) 24, (c) 48, and (d) 72 hours post-irradiation. The images in this figure are of tissue from the same patient. (size bars = 62.5 µm).

Healthy human volunteers were given a single dose of 2X MED of UVB radiation on the back. Biopsies were taken before (control) and at 1, 2, and 3 days after irradiation. Sections were stained with biotinylated HABP (Figure 5). At all three time-points after irradiation, the epidermis stained more extensively. In particular, the basal layer of the epidermis, which was unstained in control skin (Figure 5a), showed substantial accumulation of HA after single-dose UVB (Figures 5b,c,d). In the dermis, HA appears to decrease temporarily at 48 hours (Figure 5c) and then returned to baseline levels by 72 hours (Figure 5d).

### Single-dose UVB irradiation induces CS accumulation in human dermis in vivo

**Figure 6:**
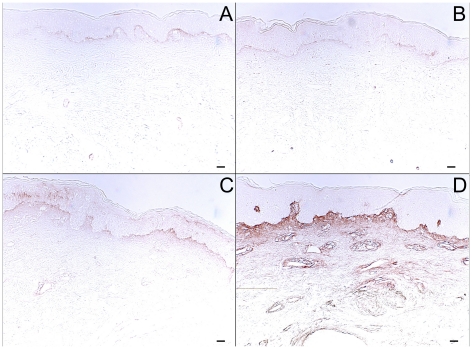
Time course for changes in CS in human skin following single-dose UVB irradiation. Normal human volunteers were exposed to 2X MED of UVB. Biopsies were taken 1, 2, and 3 days after irradiation. CS was labeled with the CS-56 anti-CS antibody and visualized using a biotin-peroxidase system. Shown are stains of (a) control tissue and tissue taken (b) 24, (c) 48, and (d) 72 hours post-irradiation. The images in this figure are of tissue from the same patient. (size bars = 62.5 µm).

In unexposed skin, dermal CS is located only in the region just below the dermal-epidermal junction and in the endothelium of dermal venules (Figures 3a, 6a). After single-dose UVB exposure, the quantity of dermal CS increases throughout the 24, 48, and 72 hour time-points, localized largely just below the dermal-epidermal junction (Figure 6b–d).

### Single-dose UVB irradiation of human skin in vivo induces accumulation of the CS isoforms C4S and C6S, but in different distributions

**Figure 7:**
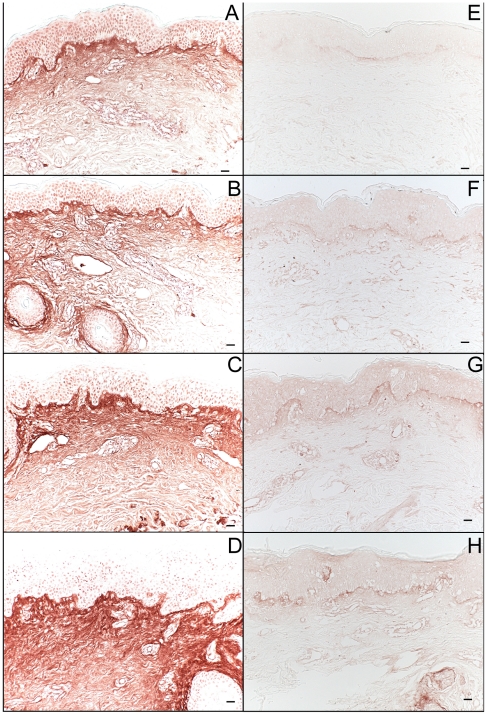
Effect of a single UVB dose on C4S and C6S in the dermis. Normal human volunteers were exposed to 2X MED of UVB. Biopsies were taken 1, 2, and 3 days after irradiation. Following enzymatic digestion, C4S was labeled using the 2B6 anti-ΔDi-4S antibody, and C6S was labeled with the 3B3 anti-ΔDi-6S antibody. The stains were developed using a biotin-peroxidase system. Shown are stains of C4S in (a) control tissue and tissue taken (b) 24, (c) 48, and (d) 72 hours post-irradiation, and of C6S in (e) control tissue and tissue taken (f) 24, (g) 48, and (h) 72 hours post-irradiation. The images in this figure are of tissue from the same patient. (size bars = 62.5 µm).

In mammals, CS exists most commonly as the 4-sulfated isoform or the 6-sulfated isoform [17]. Here, we performed immunohistochemistry for the C4S isoform on skin sections using the 2B6 anti-Δdi-4S monoclonal antibody. We found that C4S was distributed throughout the upper dermis in normal skin (Figure 7a). From 24 to 72 hours after exposure to single-dose UVB, C4S accumulated in both the upper and lower dermis (Figure 7b–d). The other CS isoform, C6S, was detected using the 3B3 anti-Δdi-6S monoclonal antibody. In unexposed control skin, C6S is present just below the dermal-epidermal junction (Figure 7e). Following single-dose UVB, C6S accumulated in the upper dermis and on endothelium, with peak staining at 48 hours post-irradiation (Figure 7f–h). Studies have indicated that CS-56 antibody has a higher affinity for C6S than C4S, and also binds to only certain epitopes of CS [18], [19], providing an explanation for differential staining seen with the C4S antibody and CS-56 (Figure 6).

### UVB and IL-1α paradoxically suppress chondroitin sulfate synthase mRNAs in cultured human dermal fibroblasts

**Figure 8:**
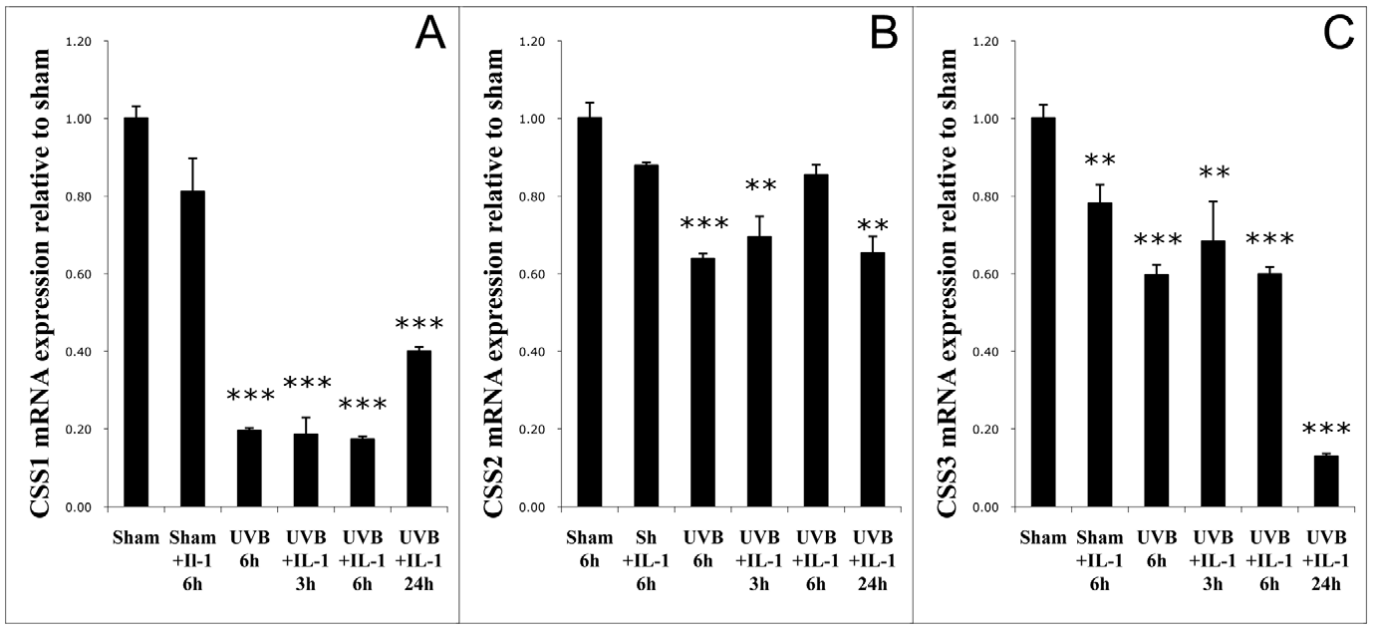
Effect of UVB +IL-1α on CSS mRNA levels in normal human fibroblasts. Human skin fibroblasts were irradiated with UVB (30 mJ/cm^2^), or sham, ± IL-1α (1 ng/ml). RNA was extracted with trizol, and CSS1 (a), CSS2 (b), and CSS3 (c) mRNA levels were evaluated using real-time PCR. (***p<.001, **p<.01).

Chondroitin polymerization is achieved by combinations of the three chondroitin sulfate synthases, CSS1, CSS2, CSS3, and chondroitin-polymerizing factor [20]. To investigate their regulation, we simulated the effects of UVB irradiation in vivo by co-stimulating cultured dermal fibroblasts with UVB (30 mJ/cm^2^) and IL-1α (1 ng/mL), a cytokine that is released from keratinocytes and inflammatory cells in skin upon exposure to UVB. RNA was extracted 3, 6, and 24 hours after treatment. Levels of mRNA for the CSS enzymes were quantified with real-time PCR. Despite the increase in dermal CS seen after UVB irradiation in vivo, CSS1 mRNA expression was significantly decreased at 3, 6, and 24 hours post-treatment, to 20-40% of control (Figure 8a). CSS2 mRNA showed modest, but significant, suppression at all time-points 3 and 24 hours post-treatment (Figure 8b). Real-time PCR revealed a steady decrease in CSS3 mRNA over the time course, with the treatment suppressing mRNA by 87% at the 24-hour time point (Figure 8c).

### Single-dose UVB irradiation induces dermal serglycin, but not decorin or versican, and UVB and IL-1α together induce serglycin mRNA in cultured human dermal fibroblasts

**Figure 9:**
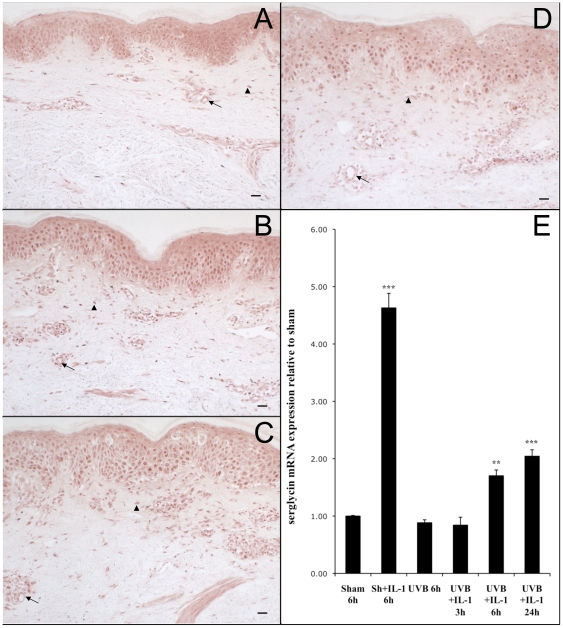
Induction of serglycin in human skin following single-dose UVB irradiation and in normal human fibroblasts following stimulation with UVB +IL-1α. Normal human volunteers were exposed to 2X MED of UVB. Biopsies were taken 1, 2, and 3 days after irradiation. Following enzymatic digestion of CS, serglycin was labeled with the N13 anti-serglycin antibody and visualized using a biotin-peroxidase system. Shown are stains of (a) control tissue and tissue taken (b) 24, (c) 48, and (d) 72 hours post-irradiation (size bars = 62.5 µm). For each, an example of the endothelium of dermal venules is indicated with an arrow, and an example of a serglycin-positive hematopoietic cell is indicate with an arrow head. The images in this figure are of tissue from the same patient. Human skin fibroblasts were irradiated with UVB (30 mJ/cm^2^), or sham, ± IL-1α (1 ng/ml). RNA was extracted with trizol, and serglycin mRNA levels were evaluated using real-time PCR (e). (***p<.001, **p<.01).

We next examined the core proteins of CSPGs known to be present in normal skin, to determine which ones might be responsible for UVB-induced CS accumulation. Stains for two major CSPGs of skin, decorin and versican, revealed no significant changes as a result of intermediate-term UVB irradiation (data not shown). In unirradiated control skin (Figure 9a), the monoclonal anti-serglycin antibody stained the epidermis, the endothelium of dermal venules, and hematopoietic cells. After single-dose UVB exposure, diffuse upper-dermal staining for serglycin steadily increased from 24 to 72 hours post-irradiation (Figure 9b–d). Dermal venule endothelium staining intensified at 24 hours post-irradiation and returned to baseline by 72 hours after exposure. The number of serglycin-expressing cells in the dermis increased with time, peaking 48 hours after exposure. The darkly stained dermal cells correspond to inflammatory cells detected by hematoxylin and eosin staining. Dermal fibroblasts were co-stimulated with UVB and IL-1α, as above, and serglycin mRNA was quantified at 3, 6, and 24 hours after treatment. Treatment significantly induced serglycin mRNA at 6 and 24 hours post-exposure, with a more than 2-fold upregulation at the 24 hour time point (Figure 9e).

## Discussion

We found that intermediate-term and single-dose UV irradiation induced highly specific responses in the glycosaminoglycans and proteoglycan core proteins of human skin. Intermediate-term UV irradiation, before the development of solar elastosis, caused dermal accumulation of CS, but not of HA, DS, or HS. We found that single-dose UV irradiation also induced dermal accumulation of CS, and two CS isoforms, C4S and C6S, were induced in distinct distributions. C4S accumulated largely in the dermis, and C6S largely in the endothelium. Regarding CS proteoglycan core proteins, UV irradiation induced the accumulation of serglycin, but not of decorin or versican, and the effect on serglycin could be mimicked in vitro by exposing human dermal fibroblasts to UVB in combination with IL-1α, a UV-induced cytokine. Serglycin was reported to have C4S side-chains when expressed by lymphocytes, platelets, and monocytes [21], and fibroblasts [22] have serglycin, but it has C6S side-chains when expressed by endothelial cells [22]. Thus this molecule could contribute to the accumulation of both C4S in dermis and C6S in endothelium after UVB exposure.

Recent work indicates that CS may ameliorate the responses to other inflammatory stimuli [23], [24]. There is evidence that CS mitigates the inflammatory effects of osteoarthritis [25]. This may be because it inhibits interleukin-1β induced pro-inflammatory cascades, as shown in cultured rabbit and human chondrocytes [26], [27]. Moreover, C4S blunts cellular activation by endotoxin [23] and by small HA fragments [28]. One study that examined C4S in the absence of other stimuli found activation of immune cells [14], suggesting that the function depends on the context.

Regarding structural effects, recent studies demonstrated that CS can inhibit elastogenesis [29], [30]. Proteins involved in elastic fiber synthesis, including fibrillin and DANCE, are strongly decreased after addition of C4S and after addition of C6S to cultured fibroblasts [30]. Because CS accumulates after intermediate-term UV exposure (Figure 3), short-term UV exposure (Figure 6), and in late-stage, solar elastotic lesions [4], its early and sustained induction may contribute to the lack of normal elastic fibers in chronically irradiated skin.

Surprisingly, although UVB increased CS in human skin, we found that mRNA levels for CSS1, CSS2, and CSS3 were paradoxically suppressed by UVB with IL-1α treatment of fibroblasts in vitro. The same stimuli increased mRNA for the core protein serglycin, suggesting that CS accumulation is due in part to increased CS core protein expression. It has been shown that the overexpression of a PG's core protein can lead to an increase in side chain generation, as overexpression of ryudocan, an HS PG, led to increased HS production [31].

Until recently, serglycin expression was thought to be limited to hematopoietic cells. Serglycin is secreted by monocytes and macrophages in response to inflammatory stimuli [21], and serglycin may also regulate the release of histamine and serotonin from mast cell granules [32]. Nevertheless, serglycin expression has been described in chondrocytes [33], and low levels of serglycin were detected in fibroblasts [22]. In the current study, we found considerable levels of serglycin expression in dermal fibroblasts after UV irradiation, suggesting that fibroblast-synthesized serglycin may be a participant in dermal inflammation. In endothelial cells, serglycin mRNA is induced by the inflammatory cytokines TNF-α and IL-1α, suggesting that it may participate in vascular inflammation as well [22].

Regarding HA, a study by Averbeck et al demonstrated that UVB increased epidermal HA and decreased dermal HA, and microdialysis revealed an accompanying accumulation of HA degradation products in the dermis [10]. Likewise, the reduced dermal staining we observed at 48 hours post-irradiation could be due to degradation of HA. Of note, fragmented HA has different biologic effects than high molecular weight HA. High molecular weight HA protects against inflammatory and apoptotic signaling in UVB-exposed corneal epithelial cells [34], and protects against angiogenesis and immune response [15]. In contrast, fragmented HA is potently pro-inflammatory and pro-angiogenic. HA fragments bind to Toll-like receptor 2 and Toll-like receptor 4 on macrophages, stimulating the expression of pro-inflammatory genes [35], as well as mediating signaling through CD44 in tissue inflammation [36]. Fragmented HA can also activate immunocompetent keratinocytes through TLR-2 and TLR4, causing the release of beta-defensin [37]. Intermediate-term and single-dose UVB exposure and intermediate-term SSR irradiation induce HA accumulation in the basal layer of the epidermis (Figures 2, 4, and 5). Further study on the molecular weight and etiology of the HA that is induced in the basal layer could elucidate its function.

Overall, our findings provide a more complete picture of the cutaneous response to ultraviolet light, where specific glycosaminoglycans and proteoglycan core proteins are likely contributors to cutaneous photodamage and photoaging.

## Materials and Methods

### Chemicals

Control, non-immune mouse IgG_1_ antibody was from Becton Dickinson. All other chemicals were obtained from Fisher (Pittsburgh, PA) and Sigma (St. Louis, MO).

### Light Sources and Radiometry

In vivo irradiation was performed using an 150-watt xenon arc solar simulator (Model #16S, Solar Light Company, Phil, PA.) with a UVB band-pass filter. For in vitro work, the UVB source was a bank of two FS-40 sunlamps (Lights of America, Walnut, CA), with a peak irradiance of 313 nm, equipped with a cellulose triacetate filter to remove wavelengths below 290 nm, as previously described [38], [39]. The light source for UVA and SSR treatments was a 1000-Watt xenon lamp solar simulator (Solar Light, Philadelphia, PA), which was used with a UG5 internal filter and an external UG11 filter to remove long wavelengths and a 3-mm WG335 Schott (UVA) filter to allow only longer UV wavelengths [39]. UVB doses were measured with an International Light UV IL-443 UVB meter. UVA and UVA1 doses were verified with an IL 1400 A Research Radiometer (International Light, Inc., Newburyport, MA). The filtered UVB light source measured by spectroradiometric measurement at the time of the experiments showed 0.64% UVC, 44.51% UVB, 19.43% UVA, and 35.42% visible and near infrared (Vis + NIR). The solar simulator with WG335 Schott filter showed 0.0036% UVC, 0.016% UVB, 96.63% UVA (11.28% UVA2 and 88.72% UVA1), 3.35% Vis + NIR.

### UV Exposure Protocols

For investigation of chronic irradiation, five human volunteers with type II skin received UVB, UVA, UVA1, SSR, PUVA, and sham irradiation to 1 cm^2^ spots of sun-protected back skin five days a week for four weeks. For PUVA treatment, .005% psoralen gel was applied topically, followed by 1X MED of UVA fifteen minutes later. Skin was biopsied 24 hours after the last irradiation, and fixed in formaldehyde. A single-dose time course was also conducted. Each patient was given a panel of eight 1 cm^2^ spots of UVB radiation in increasing increments of 25% to determine the MED, the smallest amount of UVB radiation needed to produce confluent erythema. MED testing was performed on photoprotected areas of the mid to lower back. Volunteers were exposed to a single 2X MED dose of UVB, and skin was sampled and fixed with formaldehyde 24, 48, and 72 hours post-irradiation. Fixed tissue was embedded in paraffin wax, and sections were cut onto slides for chemical stains and IHC. Human skin fibroblasts were irradiated with UVB (30 mJ/cm^2^), or sham, ± IL-1α (1 ng/ml).

### Skin biopsy staining

Biopsies were stained for uronyl-containing acid mucopolysaccharides using a modification of Mowry's colloidal iron stain (Hale stain). Staining to evaluate hyaluronan was done with biotinylated HABP (0.5 mg/mL, Associates of Cape Cod, East Falmouth, MA) used at a dilution of 1∶100 for chronically irradiated skin samples and 1∶250 for the single dose time course. Negative controls were achieved by pre-treatment of sections first with 100mM Na acetate buffer pH 5 for 15 minutes in a 37°C water bath and then with 100 TRU/ml *Streptomyces hyalurolyticus* hyaluronidase (Sigma, St. Louis, MO) in 100mM Na acetate buffer pH 5 prior to staining. A monoclonal mouse antibody for HS was purchased from Associates of Cape Cod (East Falmouth, MA) and used at a dilution of 1∶400. Negative controls with mouse IgM isotype control antibody (Sigma, St. Louis, IL) were performed concurrently. CS was evaluated using the monoclonal mouse antibody CS-56 (Sigma, St. Louis, IL), which binds chondroitin A and chondroitin C. CS-56 antibody was used at a dilution of 1∶400 to stain the intermediate-term irradiated skin sections and at a dilution of 1∶1200 on the skin that had received a single dose of UVB. Note that a lower anti-CS antibody concentration was used on the singly-irradiated sections. Binding specificity was confirmed using negative control sections that were digested with 0.025 unit/mL *Arthobacter aurescens* chondroitinase AC in a 50mM acetate buffer for 90 minutes at pH 6 prior to staining. For DS staining, sections were pre-treated with 20 mM Tris HCl buffer with 50 mM NaCl, 4 mM CaCl, and 0.01% BSA for 20 minutes at pH 7.5 and room temperature. Tissue was then digested at room temperature with *Flavobacterium heparinum* chondroitinase B (Sigma, St. Louis, IL) in buffer, and labeled with 2-B-6 anti-Δdi-4S antibody. The anti-serglycin antibody was N-13, a goat polyclonal IgG which binds near the N-terminus of human serglycin (Santa Cruz Biotechnology, Santa Cruz, CA), was used at a concentration of 10 µg/mL. C-6-S was detected using the 3-B-3 monoclonal mouse IgM (Associates of Cape Cod, East Falmouth, MA) at a dilution of 1∶100. Both the N-13 anti-serglycin and the 3-B-3 anti-Δdi-6S antibodies require enzymatic digestion of CS to expose their antigens. After a 15-minute pre-incubation with BSA-stabilized Tris-acetate buffer at 37°C and pH 8, samples were digested with 1 unit/mL *Proteus vulgaris* chondroitinase ABC (Sigma) in buffer. Negative controls were achieved by treating sections with buffer in place of the enzyme. C-4-S was stained using the 2-B-6 anti-Δdi-4S monoclonal mouse IgM (Associates of Cape Cod, East Falmouth, MA) at a dilution of 1∶600. Slides were pretreated with ph 6 acetate buffer for 15 minutes at 37°C, then digested with 025 unit/mL *Arthobacter aurescens* chondroitinase AC for antigen exposure prior to treatment with the primary antibody. Undigested sections were used as negative controls. LSAB+ Biotinylated Link Universal (DAKO, Carpinteria, CA) was used as the secondary antibody for all IHC stains. Antibodies for CS and HS, HABP were visualized with the immunoperoxidase method using the Dakocytomation LSAB+ HRP system from DAKO (Carpinteria, CA). DS, C-4-S, C-6-S, and Serglycin stains were developed with the NovaRED system (Vector, Burlingame, CA). Keratan sulfate, a GAG present in normal human skin, is not stained by Hale because it does not contain uronic acid. Therefore, it was not a component of the GAG increase measured by Hale staining, and was not evaluated.

### Quantitation of histology

Stain intensity was quantified using Image Pro Plus version 3.0 Software (MediaCybernetics, Bethesda, MD).

### Cultured cells

Normal human fibroblasts were obtained from the American Type Culture Collection (ATCC, Rockville, MD, catalog no. 1828X-CRL) and grown in Dulbecco's modified Eagle's medium supplemented with 10% fetal bovine serum.

### Extraction of total RNA from cultured fibroblasts

Cells at 60–90% confluency were irradiated with UVB or sham, followed by addition of cytokines. The viability of cells was measured by Trypan Blue staining of cells up to 72 hours after irradiation, and was always greater than 95%. After removal of medium, cells were washed with phosphate-buffered saline and total RNA was extracted by adding 1 ml Trizol (Invitrogen, Carlsbad, CA) directly to the dishes, followed by isopropanol precipitation and 70% ethanol wash. The RNA pellets were dissolved in DEPC-treated water. The RNA was quantified by measuring the optical density at 260 nm, and purity determined by 260/280 ratio (>1.8).

### Real-time PCR

Total RNA (2 g), from sham- or UVB-exposed human fibroblasts, with or without cytokine addition, was used for cDNA synthesis. cDNA was synthesized using SUPERSCRIPT First-Strand Synthesis for reverse transcription–PCR kit with random hexamers (Invitrogen Life Technologies, Carlsbad, CA). Real-time PCR was performed using Taqman primers for CSS1, CSS2, CSS3, and serglycin (product numbers Hs00208704_m1, Hs00226041_m1, Hs00545664_m1, and Hs01004159_m1 respectively from Applied Biosystems, Foster City, CA). Gene expression levels were measured by real time PCR using ABI Prism 7000 sequence detection system and normalized to GAPDH (Taqman assay: Hs99999905_m1) using ABI Prism SDS 7000 software, version 1.0 (Applied Biosystems). Each experiment was performed in triplicate in a 25 µl reaction volume in TaqMan Universal PCR Master Mix (Applied Biosystems). Each sample underwent initial denaturation for 2 minutes at 50°C and then 10 minutes at 95°C, followed by 40 cycles of denaturation at 95°C for 15 seconds and combined primer annealing and extension at 60°C for 1 minute.

### Statistics

Comparisons of several groups simultaneously were performed by initially using analysis of variance (ANOVA). When the ANOVA indicated differences amongst the groups, pairwise comparisons of each experimental group *versus* the control group were performed using the Dunnett *q*' statistic. Unless otherwise indicated, summary statistics are reported as means ± SEM, n = 4. Absent error bars in graphical displays of summary statistics indicate SEM values smaller than the drawn symbols.

### IRB approval

Detailed written informed consent was obtained from all patients, following the Declaration of Helsinki protocols. These studies were performed in accordance with a protocol that was approved by the University of Pennsylvania Institutional Review Board.
